# *In vivo *imaging of the airway wall in asthma: fibered confocal fluorescence microscopy in relation to histology and lung function

**DOI:** 10.1186/1465-9921-12-85

**Published:** 2011-06-23

**Authors:** Ching Yong Yick, Jan H von der Thüsen, Elisabeth H Bel, Peter J Sterk, Peter W Kunst

**Affiliations:** 1 Department of Respiratory Medicine, Academic Medical Centre, Meibergdreef 9, Amsterdam, The Netherlands; 2 Department of Pathology, Academic Medical Centre, Meibergdreef 9, Amsterdam, The Netherlands; 3 Department of Histopathology, Royal Brompton and Harefield NHS Foundation Trust, Sydney Street, London, UK

**Keywords:** Asthma, Confocal Laser Scanning Microscopy, Extracellular Matrix, Respiratory Function Tests, Smooth muscle

## Abstract

**Background:**

Airway remodelling is a feature of asthma including fragmentation of elastic fibres observed in the superficial elastin network of the airway wall. Fibered confocal fluorescence microscopy (FCFM) is a new and non-invasive imaging technique performed during bronchoscopy that may visualize elastic fibres, as shown by *in vitro *spectral analysis of elastin powder. We hypothesized that FCFM images capture *in vivo *elastic fibre patterns within the airway wall and that such patterns correspond with airway histology. We aimed to establish the concordance between the bronchial elastic fibre pattern in histology and FCFM. Second, we examined whether elastic fibre patterns in histology and FCFM were different between asthmatic subjects and healthy controls. Finally, the association between these patterns and lung function parameters was investigated.

**Methods:**

In a cross-sectional study comprising 16 subjects (8 atopic asthmatic patients with controlled disease and 8 healthy controls) spirometry and bronchoscopy were performed, with recording of FCFM images followed by endobronchial biopsy at the airway main carina. Elastic fibre patterns in histological sections and FCFM images were scored semi-quantitatively. Agreement between histology and FCFM was analysed using linearly weighted kappa κ_w_.

**Results:**

The patterns observed in histological sections and FCFM images could be divided into 3 distinct groups. There was good agreement between elastic fibre patterns in histology and FCFM patterns (κ_w _0.744). The semi-quantitative pattern scores were not different between asthmatic patients and controls. Notably, there was a significant difference in post-bronchodilator FEV_1 _%predicted between the different patterns by histology (p = 0.001) and FCFM (p = 0.048), regardless of asthma or atopy.

**Conclusion:**

FCFM captures the elastic fibre pattern within the airway wall in humans *in vivo*. The association between post-bronchodilator FEV_1 _%predicted and both histological and FCFM elastic fibre patterns points towards a structure-function relationship between extracellular matrix in the airway wall and lung function.

**Trial registration:**

Netherlands Trial Register NTR1306

## Background

Asthma is characterized by episodic symptoms, variable airway obstruction and airway hyperresponsiveness to a variety of inhaled stimuli [1-3], and impairment of bronchodilation following deep inspiration [4,5]. The underlying pathophysiological mechanisms that lead to the observed functional changes in asthma have only partly been resolved. Airway remodelling, a process of structural changes of the airway wall seen in asthma likely impairs lung function [6,7]. This includes an increased deposition and altered organization of extracellular matrix (ECM) proteins in the lamina propria, smooth muscle layer and adventitial layer [8-10]. Evidence shows that the elastic fibres, which represent a major component of the ECM in the airway wall, are disrupted and fragmented in asthmatic patients as compared to healthy control subjects [11]. Additionally, fragmentation and a decreased amount of elastic fibres have been observed in the superficial elastin network of central airways in fatal asthma [12].

The presence and extent of airway remodelling in asthma can be visualized by histology in endobronchial biopsy specimens. However, this is not real-time and requires careful and time-consuming histological techniques. Fibered confocal fluorescence microscopy (FCFM) is a new imaging modality, representing a non-invasive method that can be used to image the microscopic structure of airway wall tissue *in vivo *during a bronchoscopic procedure [13]. The principle of this imaging method is based on the autofluorescence of endogenous or exogenous fluorophores inside the tissues after excitation by an external laser light source. The laser light is guided through a bundle of optical microfibres to the tip of the miniprobe of 1 mm in diameter, which can be inserted into the working channel of a fibreoptic bronchoscope [14]. High-quality and real-time *in vivo *morphological images or 'optical biopsies' of the airway wall are obtained by placing the tip of the miniprobe onto the airway wall surface. Another advantage of FCFM and its miniprobe is the ability to reach and therefore visualize the alveoli *in vivo *[15].

It has been shown *in vitro *that the autofluorescence spectra of proximal bronchial mucosa and elastin powder extracted from healthy human lung at an excitation laser light wavelength of 488 nm were very similar, whereas this was not the case with bronchial mucosa and collagen I gel [13]. Therefore, the autofluorescence in FCFM images at 488 nm excitation wavelength is likely to originate from the elastic fibres present in the airway wall. However, the patterns obtained by FCFM have not yet been compared with the 'gold standard', which is histology of the airway wall.

We hypothesized that FCFM images capture elastic fibre patterns within the airway wall and that these patterns are comparable to those observed by airway histology. Therefore, the first aim of the study was to investigate the agreement between semi-quantitative pattern scores between histological sections and FCFM images. The second aim was to investigate whether the patterns seen at the airway main carina in histological sections and FCFM are different between asthmatic and healthy control subjects. Finally, we examined whether these patterns are associated with lung function.

## Methods

### Design and subjects

This study had a cross-sectional design and included 16 subjects: atopic asthmatic patients (n = 8) and healthy controls (n = 8). All subjects were recruited by the department of Respiratory Medicine of the Academic Medical Centre Amsterdam using public advertisements.

The study consisted of 2 visits. During the first visit, subjects were screened for eligibility to participate according to the in- and exclusion criteria (see below). Spirometry and a methacholine bronchoprovocation test were performed. At visit 2, bronchoscopy was carried out with real-time digital recording of FCFM images and collection of endobronchial biopsy specimens.

Asthmatic subjects had controlled disease according to GINA guidelines [1]. Furthermore, they met all of the following criteria: aged 18 to 50 years; non-smoking or having stopped smoking > 12 months with ≤ 5 pack years; no exacerbations within the last 6 weeks prior to participation; steroid-naïve or having stopped steroids by any dosing route ≥ 8 weeks prior to participation; no other medication for treating asthma than the use of inhaled short-acting β2-agonists as rescue medication; airway hyperresponsiveness defined by a methacholine bronchoprovocation test with PC_20 _≤ 8 mg/mL; post-bronchodilator FEV_1 _> 70% of predicted; and atopy defined by a positive skin prick test.

Healthy control subjects met all of the following criteria: aged 18 to 50 years; non-smoking or having stopped smoking > 12 months with ≤ 5 pack years; steroid-naïve; no airway hyperresponsiveness defined by a methacholine bronchoprovocation test with PC_20 _> 8 mg/mL; and post-bronchodilator FEV_1 _> 70% of predicted. Skin prick test was performed on all healthy control subjects, but the outcome was no selection criterion.

Participants with pulmonary diseases other than asthma were excluded, as well as pregnant females. All subjects gave written informed consent prior to enrolment. This study was approved by the Medical Ethics Committee of the Academic Medical Centre Amsterdam and is registered at the Netherlands Trial Register (NTR1306).

### Lung function and allergy

Spirometry was performed using a daily calibrated spirometer according to European Respiratory Society (ERS) recommendations [16]. The methacholine bronchoprovocation test was performed according to the standardized tidal volume method [3]. All but one of the healthy control subjects didn't reach a PC_20 _at the maximum methacholine dose of 16 mg/mL, so that we also used the linear model of the dose-response slope as proposed and validated by O'Connor *et al*. [17]. Skin prick tests were performed using 12 common aeroallergen extracts according to the position paper by the European Academy of Allergology and Clinical Immunology (EAACI) [18].

### Bronchoscopic procedure

Fibreoptic bronchoscopy was performed according to the recommendations made by the National Heart, Lung, and Blood Institute (NHLBI) and the National Institute of Allergy and Infectious Diseases (NIAID) [19]. Participants received local anaesthetic by Lignocaine 1% and 10% spray in the nose and throat. Instillation of Lignocaine 1% solution in separate lung segments was applied to further dampen the cough reflex. The oxygen saturation and heart rate of the participant were monitored continuously during the bronchoscopic procedure. Additional oxygen through an intranasal catheter was given to the participant when necessary.

**Figure 1:**
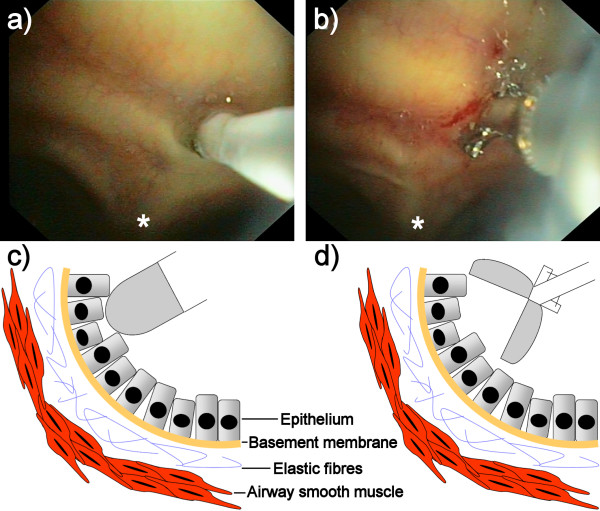
**FCFM and biopsy during bronchoscopy**. The FCFM probe was placed perpendicularly to the surface of the main carina (a) followed by endobronchial biopsy at the same location (b). Figure 1c and 1d gives a lateral view of the probe and biopsy location respectively. * = main carina.

Immediately after adequate local anaesthetic the bronchial tree was inspected with an autofluorescence bronchoscope (SAFE 3000, Pentax, Japan). Next, the Alveoflex miniprobe of the FCFM system (Cellvizio, Mauna Kea Technologies, France [20]) was inserted through the working channel of the bronchoscope and placed on the main carina of the airways. Special care was taken to position the miniprobe perpendicularly to the surface of the main carina as much as possible in order to get good quality FCFM images (Figure 1a, c). Real-time digital video recordings of 9 frames per second at 488 nm laser light excitation wavelength were made during several seconds and stored digitally. After recording the FCFM images, one endobronchial biopsy specimen was taken with a cup forceps (Pentax KW2411S) at the exact same location where the miniprobe had been placed before (Figure 1b, d). Directly after collection, the biopsy specimen was fixed in 4% buffered formaldehyde and embedded in paraffin.

### Elastic fibres and FCFM pattern analysis

Biopsy specimens were processed as described previously [21]. Briefly, biopsy specimens were cut into 4 μm sections and stained with haematoxylin and eosin for initial analysis of the bronchial morphology. One representative slide per biopsy specimen with good morphologic quality including epithelial cells, an intact reticular basement membrane, and submucosa without crushing artefacts was stained for elastin with Elastica-van Gieson (EVG). Next, images of the histological sections were captured with a digital camera coupled to a light microscope (Leica Microsystems, Germany) and analysed at 20 × magnification using Image-Pro Plus 5 (Media Cybernetics, Bethesda, MD, USA). For each slide, a representative area with positive staining for elastin in the subepithelial layer was selected for elastic fibre pattern analysis.

FCFM digital video recordings were analysed using the image analysis software MedViewer (Mauna Kea Technologies, France). This software allows detailed analysis of recordings frame by frame. Additionally, video mosaicing techniques were applied to reconstruct a FCFM digital video recording compensated for the rigid and non-rigid deformations due to motion and irregular contact of the miniprobe with the tissue surface respectively [22]. A representative image frame for each FCFM recording, in which the FCFM pattern did not change in several consecutive frames and without imaging artefacts or overexposure, was selected for pattern analysis.

**Figure 2:**
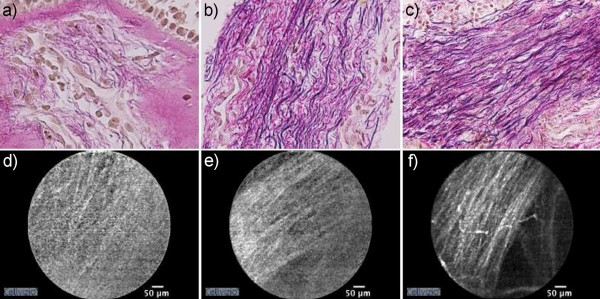
**Representative histological sections (20 × magnification) and corresponding FCFM images**. Fibres were thin and loosely organized in the 'wispy' group (a), whereas these were thick and linearly organized into a layer in the 'lamellar' group (c). No specific pattern was present in the FCFM images of the 'wispy' group (d). Individual thick lines in layer-form were observed in FCFM images of the 'lamellar' group (f). Patterns of the 'mixed' group were a combination of those seen in the 'wispy' and 'lamellar' groups (b, e).

The elastic fibre pattern in the histological sections and the FCFM patterns of the selected frames from the same subject were scored semi-quantitatively by two separate observers, who were blinded for the study groups. To define the scoring, slides stained for initial analysis of morphologic quality, were analysed. Three distinct patterns of elastic fibres were distinguished based on their thickness and organisation: wispy (score 1), mixed (score 2), and lamellar (score 3) (Figure 2). Histological sections with the classification 'wispy' contained typically thin and loosely organised elastic fibres in the subepithelial area, whereas those observed for 'lamellar' were thick and linearly organized. Additionally, the thickened fibres of the latter group were abundantly present and organized into a distinctive layer beneath the epithelium compared to the 'wispy' group. The 'mixed' group contained a mix of thin and thick elastic fibres, partly loosely and partly linearly organized.

### Data analysis

Demographic data of the study groups with a normal distribution were compared using unpaired t-tests. When normality was not achieved, data were compared using Mann-Whitney U tests. Chi-square tests were performed to analyze the distribution of the classification scores in asthmatics and controls. To analyze the agreement between semi-quantitative classification scores of the elastic fibre pattern in histology and FCFM pattern, a weighted kappa κ_w _with linear weights was calculated. A p-value of < 0.05 was considered statistically significant. The sample size of the present study was based on the estimation that a kappa of 0.8 could be detected with a power of 80% at the 5% level of significance with a study population of 14 subjects [23,24]. Statistical analyses were performed using SPSS version 18 (IBM Corporation, Somers, NY, USA).

## Results

### Subjects

Fourteen out of the total 16 test subjects were included for pattern analysis by histology and FCFM. Two subjects, including 1 asthmatic patient and 1 control subject, were excluded from analysis due to instability and/or overexposure of the FCFM images.

Subject characteristics of the study groups can be found in Table 1. The FEV_1_/FVC ratio was significantly lower and the methacholine dose-response slope significantly higher in asthmatic subjects when compared to healthy controls, as expected.

**Table 1:** Subject characteristics of asthma (A) and healthy (H) subjects

	A	H		
		
		Total	HA	HNA
**Subjects (n)**	8	8	5	3

**Male/Female (n)**	2/6	4/4	2/3	2/1

**Age (years)^a^**	24 (2)	28 (10)	30 (12)	23 (2)

**FEV_1_/FVC (%pred.)^a^**,*	96 (9)	108 (7)	105 (7)	113 (4)

**Post-bronchodilator FEV_1_** **(%pred.)^a^**	108 (11)	115 (6)	115 (6)	114 (6)

**Dose response slope (% decline FEV_1_/μmol methacholine)^b^**,**	6.49 (3.47-31.30)	0.20 (0.04-0.30)	0.23 (0.10-0.49)	0.07 (0.03-0.23)

Total = all healthy control subjects; HA = healthy, atopic; HNA = healthy, non-atopic

^a ^Mean (SD)

^b ^Median (P25-P75)

* A vs. Total p = 0.012; A vs. HNA p = 0.016

** A vs. Total p = 0.001; A vs. HA p = 0.003; A vs. HNA p = 0.014

### Elastic fibre pattern in histology

**Figure 3:**
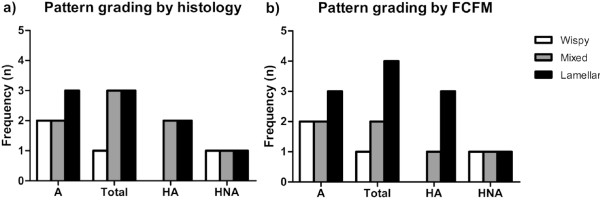
**Pattern grading by histology (a) and FCFM (b)**. A = asthma; Total = all healthy control subjects; HA = healthy, atopic; HNA = healthy, non-atopic. Chi-square test: p > 0.05.

Elastic fibres were clearly visible in the EVG-stained histological sections from all subjects. Two asthmatics and 1 healthy control subject were scored as 'wispy', whereas 2 asthmatics and 3 healthy control subjects as 'mixed'. The 'lamellar' group consisted of 3 asthmatics and 3 healthy control subjects (Figure 3a). There was no difference in histological elastic fibre patterns between asthmatic patients and healthy control subjects (p > 0.05).

### FCFM pattern

FCFM digital video recordings were classified into the three groups as described in the Methods section. In the FCFM images that were scored as 'wispy', no specific pattern could be distinguished (Figure 2). Occasionally, some indefinite and thin lines without a specific orientation could be identified. In contrast, a linearly orientated pattern composed of clearly discernible light-coloured and thick individual lines could be discriminated in FCFM images with score 3 ('lamellar'). These lines were organized into layers similar to the histological sections. The linear and orientated pattern was less clearly defined in the 'mixed' group and was a combination of the patterns seen in the 'wispy' and 'lamellar' groups. Furthermore, this 'mixed' pattern showed inter-individual variability with some resembling the 'wispy' pattern, whereas others the 'lamellar' pattern. Two asthmatics and 1 healthy control subject were scored as 'wispy', whereas 2 asthmatics and 2 healthy control subjects as 'mixed'. The 'lamellar' group consisted of 3 asthmatics and 4 healthy control subjects (Figure 3b). There was no difference in FCFM patterns between asthmatic patients and healthy controls (p > 0.05).

### Agreement between histology and FCFM

Of the 14 subjects that were included for pattern analysis, 11 subjects were scored consistently for elastic fibre pattern between histology and FCFM (weighted kappa κ_w _0.744, Table 2 and 3). There was only discrepancy between histology and FCFM in the classification of the patterns into scores 2 or 3 ('mixed' or 'lamellar').

**Table 2:** Elastic fibre pattern grading: Paired comparison histology and FCFM

Subject	Study group	Histology	FCFM
**1**	Asthma	Wispy	Wispy

**2**	Asthma	Wispy	Wispy

**3**	Asthma	*Mixed*	*Lamellar*

**4**	Asthma	Mixed	Mixed

**5**	Asthma	Lamellar	Lamellar

**6**	Asthma	*Lamellar*	*Mixed*

**7**	Asthma	Lamellar	Lamellar

**8**	Healthy, atopic	Mixed	Mixed

**9**	Healthy, atopic	*Mixed*	*Lamellar*

**10**	Healthy, atopic	Lamellar	Lamellar

**11**	Healthy, atopic	Lamellar	Lamellar

**12**	Healthy, non-atopic	Wispy	Wispy

**13**	Healthy, non-atopic	Mixed	Mixed

**14**	Healthy, non-atopic	Lamellar	Lamellar

Subjects with discrepancy between pattern scores by histology and FCFM in *Italic*.

**Table 3:** Elastic fibre pattern grading: Agreement histology and FCFM

		Histology		
		
		Wispy	Mixed	Lamellar
	Wispy	3	-	-
	
**FCFM**	Mixed	-	3	1
	
	Lamellar	-	2	5

κ_w _(SE, 95% CI) = 0.744 (0.145, 0.46-1)

### Association between patterns and lung function

**Figure 4:**
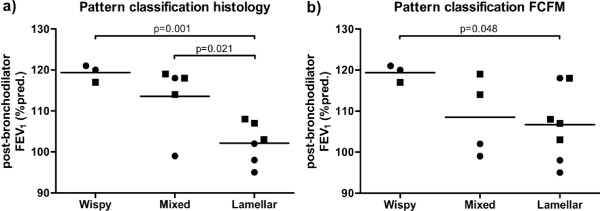
**Post-bronchodilator FEV_1 _%predicted of the three classification scores in histology (a) and FCFM (b)**. Data presented as post-bronchodilator FEV_1 _%predicted of individual subjects and the mean per classification score. ● = asthma, ■ = healthy.

Post-bronchodilator FEV_1 _%predicted was significantly lower for FCFM score 3 ('lamellar') as compared to FCFM score 1 ('wispy') (p = 0.048, Figure 4b), regardless of asthma or atopy. This was confirmed and extended by histology, showing a significantly lower post-bronchodilator FEV_1 _%predicted in the 'lamellar' as compared to the 'wispy' (p = 0.001) and 'mixed' (p = 0.021) groups (Figure 4a).

## Discussion

The present study shows that elastic fibres in the airway wall can be visualized by FCFM, a novel bronchoscopic imaging modality, and that a laminar pattern of these fibres is associated with reduced lung function. The elastic fibres in histology and FCFM images exhibited 3 distinct patterns. There was good agreement in semi-quantitative pattern score between histology and FCFM, but there were no differences in such patterns between asthma patients and controls. These findings indicate that FCFM can be used to capture structural changes in the airway wall in humans *in vivo*, and might become a real-time imaging tool to estimate the type and degree of airway remodelling in chronic airway diseases such as asthma in the near future. Since FCFM has the ability to visualize the airway wall of the whole bronchial tree *in vivo *during bronchoscopy, it has complementary advantages as compared to taking snapshot biopsies at several locations.

There are some study data and case reports in literature examining the association between FCFM and histology of e.g. preinvasive bronchial lesions and sarcoidosis [13,25]. However, to our knowledge this is the first study to investigate the histological substrate of autofluorescence in 488 nm FCFM images using *in vivo *human endobronchial biopsy specimens obtained from asthmatic patients and healthy control subjects with histology as 'gold standard'. The agreement of elastic fibre patterns between histology and FCFM is a novel finding and extends previous *in vitro *observations showing that bronchial mucosa and elastin powder extracted from human lung had similar autofluorescence spectra, suggesting that the autofluorescence in FCFM mainly originates from the elastic fibres present in the airway wall [13]. We did not observe differences in elastic fibre patterns between asthmatics and controls. This contrasts previous studies using histology [11,12], which is likely explained by differences in disease severity of the asthmatic patients, which in our study included mild disease.

The association between the elastic fibre patterns and lung function is a novel finding and adds to the validity of our histological and FCFM scoring. A plausible explanation for the lower FEV_1 _%predicted in the 'lamellar' group as compared to the other groups is that the parallel organisation of the thickened elastic fibres in a layer just beneath the epithelium changes airway wall mechanics and thereby FEV_1_. Airway wall mechanics are different in asthma as compared to controls [26], but additional ECM components e.g. collagen, proteoglycans, and glycoproteins are likely to contribute to this as well. Even though ECM may thicken the airway wall and thereby promoting luminal narrowing, any accompanying stiffening can stabilize the airways from collapse [27]. It is still unknown how elastic fibre patterns could influence either of the above mechanisms. Hence, our current structure-function observations should be considered as hypothesis-generating.

The present results suggest that FCFM is an adequate method to examine bronchial elastic fibre morphology *in vivo *and might be an important tool to detect asthmatic patients who are prone to loss of lung function, at an early stage enabling timely intervention. Based on data found in literature we would expect that the degree of airway remodelling differs with asthma severity, including a varying amount or organization of elastic fibres [10,12,28-30]. Therefore, subsequent studies including larger numbers of subjects with varying asthma severity and FCFM images of multiple locations in the bronchial tree are needed. These will give a more detailed insight into the association between histology, FCFM, and airway function.

The strength of our study is that we applied strict patient selection criteria and that histology and FCFM were obtained from the same endobronchial sites. The bronchial main carina was chosen as the location for FCFM and biopsy to minimize imaging artefacts resulting from inadequate positioning of the miniprobe superimposed on the movement of the airways due to tidal breathing. However, there are potential limitations that need to be addressed. First, the number of 16 subjects was relatively low when using 3 semi-quantitative scores. Although this study was powered on kappa, power estimation based on this value is not firmly developed yet. This is due to the fact that this estimation is dependent on the kappa expected to be found and the marginal frequencies, which are the proportions of test subjects in each semi-quantitative category of histology and FCFM. Second, FCFM images during bronchoscopy were captured by placing the miniprobe perpendicularly to the surface of the airway main carina followed by a biopsy from the same location. It was technically impossible to orientate the small biopsy specimens in such a way that the cutting plane was identical to the plane of view during the FCFM recordings. This may have introduced bias in the semi-quantitative scoring of the histological sections. However, this bias seems to be limited as only the superficially located elastic fibres in the subepithelial layer were graded.

Our findings show a good agreement between pattern scores by histology and FCFM. The FCFM miniprobe has a fixed depth of view of 50 μm and therefore captures images at the level of the subepithelial layer. Accordingly, elastic fibre patterns in the subepithelial layer were scored in the histological sections. Pattern scores by histology and FCFM proved to be close in resemblance. By analysing both the autofluorescence patterns and the surrounding darker areas in FCFM images, this imaging modality also has the potential to visualize airway remodelling in general, which today is only possibly by histology of biopsy specimens. Other bronchoscopic real-time imaging modalities visualizing airway wall structures have recently been introduced including anatomical optical coherence tomography (*a*OCT) and endobronchial ultrasonography (EBUS) [31-34]. While *a*OCT and EBUS may visualize the different layers of the airway wall, FCFM can image a specific airway structural component in microscopic detail. Nevertheless, all three imaging techniques are not suitable to replace histology in the clinical setting yet. The technical part has to be further improved to acquire even higher quality images with minimal imaging artefacts.

## Conclusions

In the current study, we observed good agreement between elastic fibre pattern scores of the bronchial wall by histology and fibered confocal fluorescence microscopy, suggesting that this imaging technique is suitable to capture the morphology of bronchial elastic fibres non-invasively in humans *in vivo*. Post-bronchodilator FEV_1 _%predicted was associated with elastic fibre patterns, pointing towards a structure-function relationship between extracellular matrix and lung function. The results of our study suggest that fibered confocal fluorescence microscopy might become a real-time imaging tool to estimate the type and degree of airway remodelling in chronic airway diseases such as asthma.

## List of abbreviations

**EAACI**: European Academy of Allergology and Clinical Immunology; **EBUS**: endobronchial ultrasonography; **ECM**: extracellular matrix; **ERS**: European Respiratory Society; **EVG**: Elastica-van Gieson; **FCFM**: fibered confocal fluorescence microscopy; **FEV_1_**: forced expiratory volume in 1 second; **FVC**: forced vital capacity; **GINA**: Global Initiative for Asthma; **NHLBI**: National Heart, Lung, and Blood Institute; **NIAID**: National Institute of Allergy and Infectious Diseases; ***a*OCT**: anatomical optical coherence tomography; **PC_20_**: provocative concentration of methacholine causing a 20% drop in forced expiratory volume in 1 second;

## Competing interests

The authors declare that they have no competing interests.

## Authors' contributions

CYY carried out the study procedures and spirometry measurements, participated in the design of the study, and wrote the manuscript. JHVDT carried out the sectioning, staining, and analysis of the biopsy specimens, and helped to draft the manuscript. EHB participated in the design of the study and its coordination, and helped to draft the manuscript. PJS participated in the design of the study and its coordination, and helped to draft the manuscript. PWK conceived the study, participated in its design and coordination, performed all bronchoscopic procedures, and helped to draft the manuscript. All authors read and approved the final manuscript.
